# Association between perinatal methylation of the neuronal differentiation regulator *HES1* and later childhood neurocognitive function and behaviour

**DOI:** 10.1093/ije/dyv052

**Published:** 2015-04-22

**Authors:** Karen A Lillycrop, Paula M Costello, Ai Ling Teh, Robert J Murray, Rebecca Clarke-Harris, Sheila J Barton, Emma S Garratt, Sherry Ngo, Allan M Sheppard, Johnny Wong, Shaillay Dogra, Graham C Burdge, Cyrus Cooper, Hazel M Inskip, Catharine R Gale, Peter D Gluckman, Nicholas C Harvey, Yap-Seng Chong, Fabian Yap, Michael J Meaney, Anne Rifkin-Graboi, Joanna D Holbrook, The Epigen Global Research Consortium, Keith M Godfrey

**Affiliations:** ^1^Centre for Biological Sciences, and; ^2^Academic Unit of Human Development and Health, University of Southampton, Southampton, UK,; ^3^Singapore Institute for Clinical Sciences, Agency for Science Technology and Research, Singapore,; ^4^MRC Lifecourse Epidemiology Unit, University of Southampton, Southampton, UK,; ^5^Liggins Institute, University of Auckland, Auckland, New Zealand,; ^6^NIHR Southampton Biomedical Research Centre, University Hospital Southampton, Southampton, UK,; ^7^NIHR Musculoskeletal Biomedical Research Unit, University of Oxford, Oxford, UK,; ^8^Centre for Cognitive Ageing and Cognitive Epidemiology, University of Edinburgh, Edinburgh, UK,; ^9^Department of Obstetrics and Gynaecology, Yong Loo Lin School of Medicine, National University of Singapore, Singapore,; ^10^Department of Paediatrics, KK Women’s and Children’s Hospital, Singapore,; ^11^Duke NUS Graduate School of Medicine, National University of Singapore, Singapore,; ^12^Lee Kong Chian School of Medicine, Nanyang Technological University, Singapore and; ^13^Ludmer Centre for Neuroinformatics and Mental Health, McGill University, Montréal, Canada

**Keywords:** *HES1*, methylation, neurocognitive development, epigenetic, perinatal

## Abstract

**Background** Early life environments induce long-term changes in neurocognitive development and behaviour. In animal models, early environmental cues affect neuropsychological phenotypes via epigenetic processes but, as yet, there is little direct evidence for such mechanisms in humans.

**Method** We examined the relation between DNA methylation at birth and child neuropsychological outcomes in two culturally diverse populations using a genome-wide methylation analysis and validation by pyrosequencing.

**Results** Within the UK Southampton Women’s Survey (SWS) we first identified 41 differentially methylated regions of interest (DMROI) at birth associated with child’s full-scale IQ at age 4 years. Associations between *HES1* DMROI methylation and later cognitive function were confirmed by pyrosequencing in 175 SWS children. Consistent with these findings, higher *HES1* methylation was associated with higher executive memory function in a second independent group of 200 SWS 7-year-olds. Finally, we examined a pathway for this relationship within a Singaporean cohort (*n* = 108). Here, *HES1* DMROI methylation predicted differences in early infant behaviour, known to be associated with academic success. *In vitro*, methylation of *HES1* inhibited ETS transcription factor binding, suggesting a functional role of this site.

**Conclusions** Thus, our findings suggest that perinatal epigenetic processes mark later neurocognitive function and behaviour, providing support for a role of epigenetic processes in mediating the long-term consequences of early life environment on cognitive development.

**Key Messages**

- An association was found between umbilical cord methylation of CpG loci within the *HES1* gene, a key regulator of neuronal differentiation and brain patterning, with child’s full-scale IQ age 4 years and executive function at 7 years in two independent groups of UK children.
- Methylation of the identified CpG loci within *HES1* *in vitro* inhibited ETS transcription factor binding, suggesting a functional role of this site.
- Thus, our findings suggest that perinatal epigenetic processes mark later neurocognitive function and behaviour, providing support for a role of epigenetic processes in mediating the long-term consequences of early life environment on cognitive development.

## Introduction

There is now substantial evidence that the quality of the early life environment both before and after birth is important for later cognitive function. Birthweight,^1^^,^^2^ maternal^3^ or childhood^4^ stress and poor nutrition^5^^,^^6^ in early life have all been linked to poorer neuro-behavioural and cognitive function in later life, but to date the mechanisms mediating these affects are largely unknown.

Experimental studies suggest that the developmental environment can influence neuropsychological function through alterations in epigenetic gene regulation. Epigenetic processes such as DNA methylation can induce changes in gene expression without a change in DNA base sequence.^7^ Such processes are involved in cell differentiation and genomic imprinting, as well as the phenomenon of developmental plasticity in response to environmental influences.^8^ Through these mechanisms, early life environmental factors can affect the developmental trajectory, with long-term effects on gene expression and phenotypic outcome.^9^ For example, in rodents maternal behaviour induced stable changes in DNA methylation and histone modifications in the hippocampal glucocorticoid receptor (*GR*) gene promoter in the offspring, affecting stress responses throughout the life course.^10^ In humans, the evidence for such processes is necessarily indirect. Adult suicide victims abused as children had higher *GR* methylation in post-mortem hippocampal samples compared with suicide victims with no such history.^11^ The hippocampus is essential to both stress regulation and learning, raising the possibility that methylation changes induced in early life may affect behavioural and cognitive functioning. However, to date there have been no longitudinal studies showing that prenatal epigenetic processes are associated with childhood neurocognitive development.

Whereas many DNA methylation patterns are tissue specific, recent studies indicate that some epigenetic marks show both inter-individual variation and some equivalence between different tissue types.^12–15^ For example, a relationship between childhood adversity and *GR* methylation has been reported in both the hippocampus and in peripheral blood cells,^13^ suggesting that peripheral tissues could be used to study developmentally induced epigenetic marks associated with later neuropsychological function.

To investigate whether developmentally induced epigenetic processes relate to later cognitive function, we employed an epigenome-wide approach to identify methylation differences in umbilical cord genomic DNA that were associated with child’s cognitive performance at age 4 years. We validated the association between perinatal methylation levels of *HES1*, a gene with a pivotal role in neuronal differentiation and the formation of organising centres within the brain,^16^^,^^17^ and later cognitive function in two culturally diverse populations, demonstrating that epigenetics may mediate the long-term consequences of the early life environment on cognitive development.

## Methods

### Southampton Women’s Survey

The Southampton Women’s Survey (SWS) is a prospective mother-offspring cohort.^18^ At age 4 years, a sub-sample of participants had their full-scale IQ assessed [Wechsler Pre-School and Primary Scale of Intelligence (WPPSI-III, UK)].^19^ At 7 years, a different SWS sub-sample participated in the Cambridge Neuropsychological Test Automated Battery [CANTAB Delayed Matching to Subject (DMS), Intra-Extra Dimensional Set Shift (IED) and Spatial Span (SSP)]^20^ tests. Further details are in Supplementary Methods 1 and cohort characteristics are shown in Supplementary Table 1, available as Supplementary data at *IJE* online.

### Growing Up in Singapore Towards Healthy Outcomes (GUSTO)

In the GUSTO prospective mother-offspring cohort study,^21^ socio-emotional data were available for 108 1-year-old infants for whom umbilical cord DNA had previously been collected. Socio-emotional behaviour was assessed via maternal report using the Infant Toddler Socio-Emotional Assessment (ITSEA).^22^ The Externalising domain of this tool assesses early manifestations of socially disruptive behaviour such as aggression and defiance, linked with lower cognitive performance.^23^ Further details are in Supplementary Methods 2 and cohort characteristics are shown in Supplementary Table 2, available as Supplementary data at *IJE* online.

### Whole genome methylation analysis

Genomic DNA from SWS umbilical cord samples with later neurocognitive data at age 4 years (*n* = 24, minimum and maximum IQ for each group: Group 1 81–99, Group 2 101–107, Group 3 113–18 and Group 4 121–122) was extracted, sonicated and methylated DNA isolated using a His-tagged MBD2b (methyl-binding domain of MeCP2) protein according to the manufacturer’s instructions (MethylCollector kit, Active Motif). After methyl capture, the labelled methylated DNA and input DNA was hybridised to the Agilent Human Promoter Whole-Genome ChIP-on-chip array (G4489A; see Supplementary Methods 3, available as Supplementary data at *IJE* online) which contains probes spanning the promoter regions of 25 000 genes from −5.5 kb of the TSS to 2.5 kb downstream.

### Methylation array data analysis

The log2 of Cy5/Cy3 values was obtained for each probe after background subtraction, and processed by the Bayesian Tool for Methylation Analysis (BATMAN).^24^ Log2 ratios of tiled probes and CpG densities in the probe and 100 nt of flanking genomic sequence are assessed to calculate likely percentage methylation value distributions. The mode of the distribution for each 100 nt region returned by BATMAN was used for further analysis. Examining the frequency distribution of the BATMAN output as well as the raw log2 ratios^25^ revealed that most samples had a frequency distribution close to a beta distribution, both before and after BATMAN analysis. The peaks were mapped to the probes/genes using the Agilent identifiers.

### Identification of DMRs and DMROIs

Differentially methylated regions (DMRs) and DMROIs were identified using WPPSI data. SWS subjects were grouped into four separate ordinal categories according to WPPSI score, with Group 1 having the lowest scores and Group 4 the highest. DMRs were defined as 100 nt regions fulfilling the following criteria: (i) robust regression analysis *P* ≤ 0.02 (to correct for heteroscedasticity);^26^ (ii) Mann-Whitney test between WPSSI Group 1 vs 4 giving *P* ≤ 0.02 and *P* ≤ 0.01 for Mann-Whitney test between WPSSI Group 1 vs 2, or Group 2 vs 3 or Group 3 vs 4; (iii) MethOR ≤ 0.667 or MethOR ≥ 1.5 for WPSSI Group 1 vs 4; (iv) absolute methylation differences between WPSSI Groups 1 and 4 ≥ 20%. The Fisher Exact test was applied to test if a Region of Interest (ROI) was enriched (i.e. number of DMRs within the ROI getting *P*-value < 0.01) against the background. We calculated the proportion of DMRs with *P*-value less than 0.01 and compared it against the background. The probability was calculated as below:

$$P=\frac{\left(\begin{array}{c} a+b\\ a \end{array}\right)\left(\begin{array}{c} c+d\\ c \end{array}\right)}{\left(\begin{array}{c} n\\ a+c \end{array}\right)}$$

where a = # of DMR with *P*-value < 0.01 in the ROI, b = # of DMR with *P*-value < 0.01 in the entire data set, c = # of DMR with *P*-value ≥ 0.01, d = # of DMR with *P*-value ≥ 0.01 in the entire data set and *n* = total number of DMRs.

DMROIs were then defined as giving Fisher Exact tests *P* ≤ 0.01 for 100 nucleotide regions and Mann-Whitney *P* ≤ 0.02 between Group 1 vs 4 and containing at least one DMR. The cut-offs used to select DMRs/DMROIs were designed to be a stringent filter to prioritise genes for the pathway analysis. Pathway enrichment analysis used the MetaCoreTM network analysis suite (GeneGo Inc),^27^ with the design of the array set as the background in the pathway analysis.

### Pyrosequencing

Array methylation results were validated by sodium bisulphite pyrosequencing. Briefly, pyrosequencing assays were designed to sequence the individual CpG dinucleotides within the DMROI (primers are listed in Supplementary Table 3, available as Supplementary data at *IJE* online) using Pyromark Assay Design Software 2.0 (Qiagen, Hilden, Germany); assays were analysed (PSQ 96MA machine; Biotage, Uppsala, Sweden) and percentage methylation calculated using Pyro Q-CpG software (Biotage). The sequenced region for *HES1* encompassed only 9 of 15 CpGs in the 920 bp BATMAN DMROI, due to sequence design constraints.

### Electrophoretic mobility shift assays

Electrophoretic mobility shift assays were carried out^28^ using 5 µg of IMR32 nuclear extract (sc-2148, Santa Cruz Biotechnology, USA). Supplementary Table 3 shows oligonucleotide sequences, available as Supplementary data at *IJE* online.

### Statistical analysis of pyrosequencing data

Statistical analysis used Stata (Statacorp) versions 11.2/12.1. Pyrosequencer methylation measurements did not approximate a Normal distribution and were transformed using Fisher-Yates Normal scores with mean of zero and standard deviation (SD) of one. Regression models were built using the child’s neuropsychological measure [at 1 (GUSTO), 4 or 7 (SWS) years] as the outcome and methylation of the nine CpGs measured as the predictor, adjusted for sex and then further adjusted for sex and either mother’s IQ (4-year WPPSI) or mother’s highest educational attainment (7-year CANTAB, 1-year ITSEA) as available; our previous studies found little additional influence of socioeconomic status after controlling for mother’s IQ.^29^ Subsequently, age at assessment, birthweight, maternal smoking, BMI and parity were included as covariates. Results presented are regression coefficients (β), representing the change in neurodevelopmental outcome per SD change in percentage methylation, and associated *P-*values.

## Results

### Characteristics of the cohorts

The SWS cohort subjects (*n* = 175) with 4-year cognitive measurements (median 4.4 years) had a median birthweight of 3.5 kg (Supplementary Table 1, available as Supplementary data at *IJE* online). The 200 children from the SWS cohort with 7-year cognitive measurements (median 7.0 years) had a similar birthweight distribution with a median birthweight of 3.4 kg (Supplementary Table 1, available as Supplementary data at *IJE* online). The median maternal age at birth of the child and pre-pregnancy body mass index were similar in the SWS 4- and 7-year subjects (30.4 vs 32.2 years and 24.5 vs 24.3 kg/m^2^, respectively). The 108 children from the GUSTO cohort had a median age of 0.99 years and a birthweight of 3.09 kg (SupplementaryTable 2, available as Supplementary data at *IJE* online). The median maternal age at birth and pre-pregnancy body mass index were also similar in the GUSTO cohort (31.7 years and 25.1 kg/m^2^, respectively).

### Identification of differentially methylated regions of interest at birth associated with later cognitive performance

**Figure 1.**
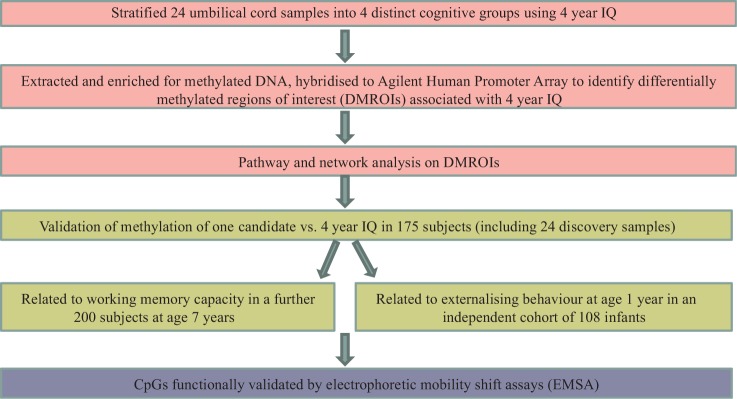
Overview of study.

**Figure 2.**
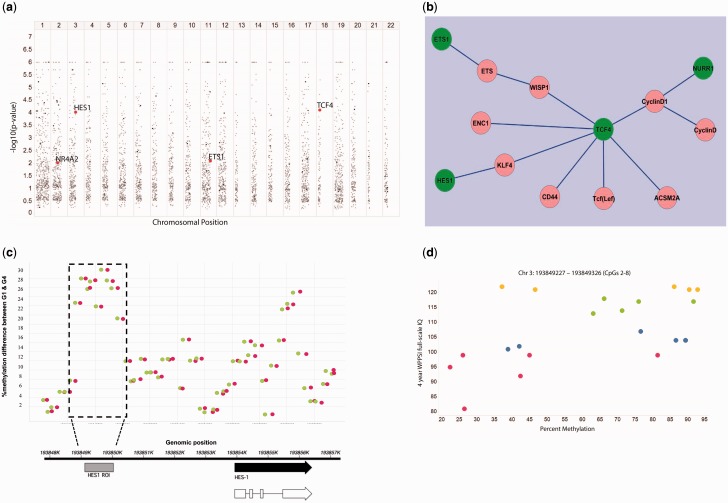
Methylation of *HES1* DMR in cord at birth is associated with WPPSI IQ at 4 years of age. a) Manhattan plot of epigenome-wide methylation analysis. The X-axis indicates chromosomal position, the Y-axis the –log 10 *P*-value of the Fishers Exact test. The black dots represent DMROIs and those associated with *HES1*, *NR4A2*, *ETS1* and *TCF4* are shown in red. b) Diencephalon development pathway. Genes contained in the diencephalon development GO process were connected to each other by using the direct interactions algorithm in GeneGoMetacore^TM^. Genes containing DMROIs (*HES1, NURR1, ETS1* and *TCF4*) are denoted by green circles (*NR4A2* is denoted by its alternative name *NURR1*). This figure is generated in Cytoscape**. c)** DMROI plot for *HES1* (Chr 3: 193848528–193853872). X-axis shows chromosomal coordinates (hg19), Y-axis shows absolute % methylation difference between WPSSI Groups 1 and 4. Green and red circles represent start and end of each 100-nucleotide region returned from BATMAN, respectively; 100 nucleotide regions in the dotted box were found to have >20% absolute methylation difference between WPSSI Groups 1 and 4 and this region was selected for pyrosequencing in the extended sample set. The lower panel shows the positions of the *HES1* transcript and the upstream DMROI. d) Concordance of methylation values with WPSSI scores for the 100-nt region within the *HES1* ROI selected for pyrosequencing (containing CpGs 2–8), upstream of the *HES1* coding sequence. X-axis shows WPSSI scores and Y-axis shows % methylation as estimated by the Bayesian algorithm BATMAN. Sample data points are coloured by WPSSI groups (red = Group 1, lowest WPSSI scores; blue = Group 2, low WPSSI scores; green = Group 3, high WPSSI scores; yellow = Group 4, highest WPSSI scores). Chromosomal coordinates of the region are detailed above the figure. WPPSI = Wechsler Pre-School and Primary Scale of Intelligence (full-scale IQ).

Genomic umbilical cord DNA from 24 SWS children was screened using the MBD array for differences in DNA methylation at birth associated with WPPSI IQ age 4 years (Figure 1). The subjects selected were representative of the range of WPPSI IQ measurements within the whole cohort. Statistical analysis of the data identified 41 DMROIs associated with IQ at age 4 years (Supplementary Table 4, available as Supplementary data at *IJE* online; Figure 2a).

### The diencephalon development process was significantly enriched for DMROIs

The top pathway enriched for DMROIs in the GO process category was diencephalon development (4/71 genes, *P* = 0.000044; Supplementary Table 5, available as Supplementary data at *IJE* online), which is important for the integration of cognitive function.^30^ Figure 2b shows a sub-network created by direct interactions between genes contained within the diencephalon development GO process and includes four genes: *HES1*, *NR4A2* (also known as *NURR1*), *ETS1* and *TCF4* which contained DMROIs. Methylation at birth within the *HES1* DMROI, as estimated by BATMAN, was positively associated with WPPSI IQ at age 4 years (Figure 2c). The associations for *NR4A2* and *TCF4* were also positive whereas the *ETS1* association was negative.

### Validation of *HES1* DMROI

**Figure 3.**
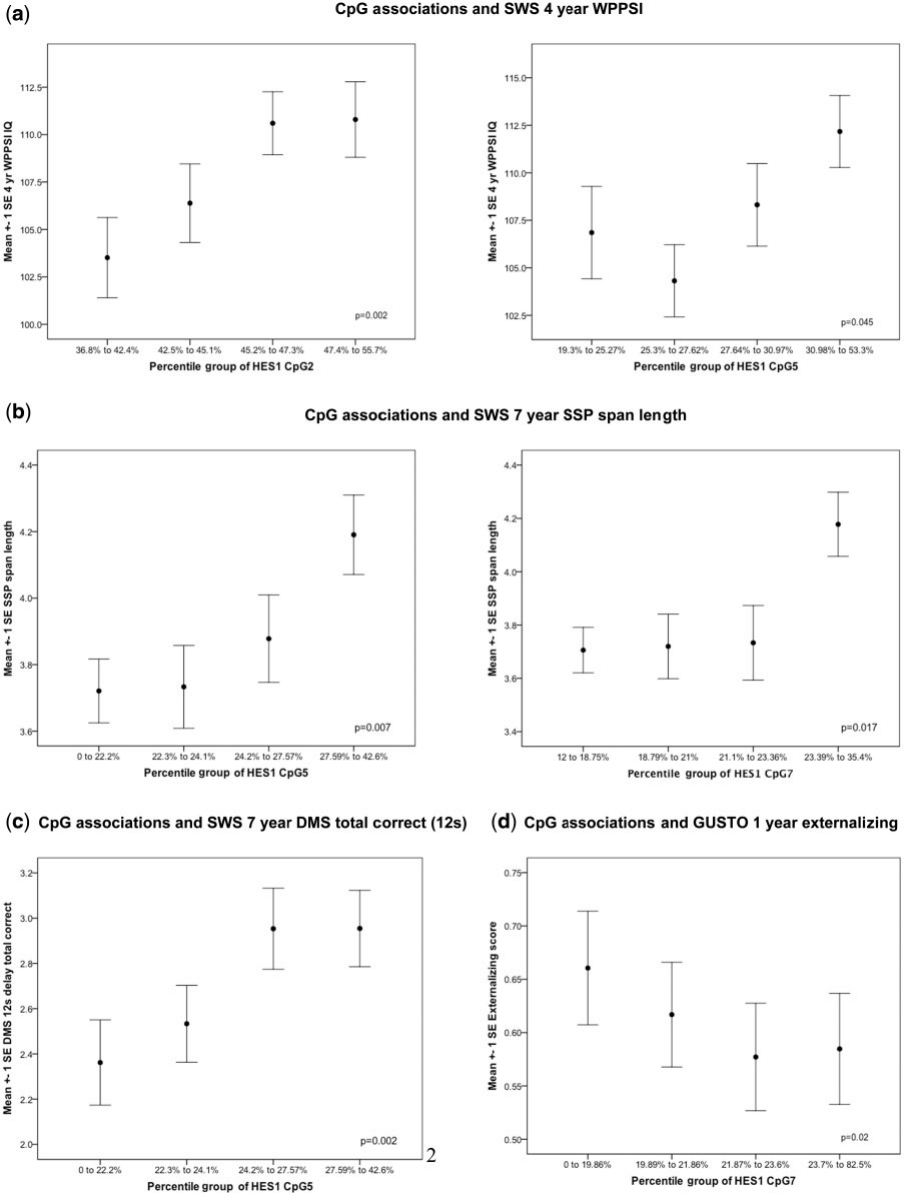
*HES1* DMROI methylation at birth is associated with childhood neuropsychological function. a) Association between cord *HES1* CpG2 and CpG5 methylation and Wechsler Pre-School and Primary Scale of Intelligence (WPPSI IQ) at age 4 years**. b)** Association between cord *HES1* CpG5 and CpG7 methylation and spatial span length at 7 years of age. c) Association between cord *HES1* CpG 5 methylation and delayed matching to sample (DMS) 12 s delay total correct at 7 years of age. d) Association between cord *HES1* CpG7 methylation and infant externalising score. Methylation has been divided into 4 equal groups according to rank; means and standard errors are plotted for each group. *P*-values are for regression of continuous variables adjusting for gender and mother’s IQ.

We chose to validate *HES1* since it has been shown to play an essential role in the generation of organising centres within the brain of the appropriate size, shape and specification by controlling the timing of cell differentiation within the CNS.^16^ Moreover, the region of *HES1* identified as a DMROI was located 4.8 kb upstream of the transcription start site (TSS), in a region of *HES1* that is evolutionarily conserved, suggesting that altered methylation of this region of *HES1* may have important functional consequences for neuronal differentiation and function. Methylation levels of nine CpGs within this region were analysed by pyrosequencing in an extended sample of 175 SWS subjects (including the 24 samples used for the MBD array) for which 4-year WPPSI data were available. The concordance of methylation values with WPPSI scores for the 100 bp region within the *HES1* ROI selected for pyrosequencing validation can be seen in Figure 2d. Consistent with these findings from the MBD array, associations were seen between the cord DNA methylation status of individual CpGs within the DMROI of *HES1* and the child’s 4-year WPPSI IQ (Figure 3a). Higher percentage methylation of CpG2 associated with higher 4-year WPPSI IQ (β = 2.693, *P* = 0.009) with a trend for CpG5 (β = 1.951, *P* = 0.072, Table 1). Adjusting for the mother’s IQ strengthened associations between the percentage methylation of CpGs 2 and 5 and child’s IQ (β = 3.192, *P* = 0.002; β = 2.140, *P* = 0.045, respectively; Table 1); omitting the original 24 discovery samples from these analyses had little effect on the associations (e.g. for CpG 2 revised, β = 3.179, *P* = 0.005). Likewise, further adjustment for maternal smoking, BMI and parity and the child’s birthweight and age at WPPSI measurement had little effect on the magnitude and statistical significance of the association with CpG2, but for CpG5 there was an attenuation (Supplementary Table 6, available as Supplementary data at *IJE* online). The multivariate model combining *HES1* CpG2, child’s IQ and maternal IQ explained 15.7% of the WPPSI variability; similar variability was explained by models replacing CpG2 with CpG5 or CpG7 methylation. The presence of SNPs at the identified CpG sites in *HES1* were excluded by direct sequencing of this region.

**Table 1.** Association of umbilical cord *HES1* CpG methylation within the identified DMROI with 4-year WPPSI outcomes in 175 children

	4-year WPPSI IQ, adjusted for child’s sex				4-year WPPSI IQ, adjusted for child’s sex and mother’s WASI					
	*n*	β	LCL	UCL	*P*-value	*n*	β	LCL	UCL	*P*-value
*HES1*
CpG1	168	1.068	−0.853	2.989	0.277	168	1.341	−0.563	3.244	0.169
CpG2	157	2.693	0.694	4.692	0.009**	157	3.192	1.212	5.173	0.002**
CpG3	154	0.260	−1.801	2.321	0.805	154	0.540	−1.510	2.589	0.606
CpG4	146	1.457	−0.582	3.495	0.164	146	1.571	−0.437	3.579	0.127
CpG5	139	1.951	−0.161	4.062	0.072*	139	2.140	0.066	4.215	0.045**
CpG6	170	0.936	−0.990	2.861	0.342	170	1.041	−0.863	2.945	0.285
CpG7	155	1.399	−0.623	3.421	0.177	155	1.719	−0.292	3.729	0.096*
CpG8	146	1.014	−1.027	3.054	0.332	146	1.501	−0.543	3.546	0.152
CpG9	138	0.928	−1.212	3.067	0.397	138	1.512	−0.661	3.684	0.175

WPPSI, Wechsler Pre-School and Primary Scale of Intelligence (full-scale IQ); WASI, *Wechsler Abbreviated Scale of Intelligence* (full-scale IQ); LCL, lower 95% confidence limit; UCL, upper 95% confidence limit.

*P-value < 0.1; **P-value ≤ 0.05.

### Association of cord *HES1* methylation with executive function at age 7 years

To determine whether the methylation status of *HES1* at birth was also associated with cognitive function at 7 years of age, methylation of the CpGs within the DMROI of *HES1* was also measured in a further subset of SWS children assessed for executive function using CANTAB. The range and average methylation of CpGs within the DMROI of *HES1* were similar in the two groups of children assessed at ages 4 and 7 years (Supplementary Table 7, available as Supplementary data at *IJE* online) and the CpGs were highly correlated for both age groups, with correlation coefficients between 0.31 and 0.85 (Supplementary Table 8, available as Supplementary data at *IJE* online). Higher *HES1* CpG 1, 5, 6 and 7 % methylation were associated with enhanced executive function, indicated by greater SSP span length (CpG5, β = 0.168, *P* = 0.007; CpG6, β = 0.135, *P* = 0.026; and CpG7, β = 0.144, *P* = 0.017; Table 2, Figure 3b) and greater DMS 12 s delay total correct (CpG1, β = 0.196, *P* = 0.028; CpG 5, β = 0.285, *P* = 0.002; CpG6, β = 0.174, *P = *0.043; and CpG7, β = 0.167, *P* = 0.052; Table 2, Figure 3c). *HES1* CpG8 methylation was associated with IED total errors (Stage 1). The positive association seen between *HES1* methylation and CANTAB measurements was in the same direction as found between *HES1* methylation and child’s IQ at 4 years of age.

**Table 2.** Association of umbilical cord *HES1* CpG methylation within the identified DMROI with 7 year CANTAB outcomes in 200 children

	**CANTAB outcomes adjusted for sex**					**CANTAB outcomes adjusted for sex and mother’s educational attainment**				
	***n***	**beta**	**LCL**	**UCL**	***P*-value**	***n***	**beta**	**LCL**	**UCL**	***P*-value**
DMS total correct										
HES1 CpG1	187	0.196	0.022	0.369	0.028**	186	0.205	0.028	0.382	0.024**
HES1 CpG2	180	0.121	−0.060	0.302	0.192	179	0.122	−0.063	0.306	0.198
HES1 CpG3	180	0.175	−0.007	0.357	0.061*	179	0.171	−0.012	0.354	0.069*
HES1 CpG4	179	0.087	−0.098	0.271	0.359	178	0.095	−0.094	0.283	0.327
HES1 CpG5	179	0.285	0.111	0.459	0.002**	178	0.289	0.111	0.467	0.002**
HES1 CpG6	200	0.174	0.007	0.342	0.043**	199	0.175	0.005	0.345	0.045**
HES1 CpG7	199	0.167	−0.001	0.334	0.052*	198	0.164	−0.007	0.334	0.061*
HES1 CpG8	197	0.151	−0.017	0.319	0.079*	196	0.152	−0.02	0.324	0.085*
HES1 CpG9	197	0.097	−0.072	0.266	0.261	196	0.097	−0.075	0.268	0.272
IED total errors (Stage 1)										
HES1 CpG1	185	0.023	−0.150	0.196	0.797	184	0.024	−0.153	0.201	0.793
HES1 CpG2	178	0.143	−0.034	0.321	0.115	177	0.142	−0.039	0.322	0.125
HES1 CpG3	178	0.128	−0.051	0.308	0.163	177	0.123	−0.058	0.303	0.185
HES1 CpG4	177	0.155	−0.025	0.335	0.093*	176	0.165	−0.020	0.349	0.082*
HES1 CpG5	177	0.070	−0.106	0.245	0.437	176	0.063	−0.116	0.243	0.492
HES1 CpG6	198	0.161	−0.007	0.330	0.062*	197	0.161	−0.010	0.332	0.066*
HES1 CpG7	197	0.095	−0.073	0.263	0.269	196	0.091	−0.080	0.263	0.298
HES1 CpG8	195	0.195	0.028	0.363	0.023**	194	0.198	0.026	0.369	0.025**
HES1 CpG9	195	0.149	−0.020	0.318	0.086*	194	0.148	−0.024	0.320	0.092*
IED total errors (Stage 8)										
HES1 CpG1	185	−0.007	−0.213	0.199	0.948	184	−0.013	−0.224	0.197	0.901
HES1 CpG2	178	−0.089	−0.296	0.118	0.402	177	−0.088	−0.299	0.122	0.412
HES1 CpG3	178	−0.092	−0.302	0.117	0.388	177	−0.087	−0.297	0.124	0.421
HES1 CpG4	177	0.134	−0.076	0.344	0.213	176	0.131	−0.084	0.346	0.234
HES1 CpG5	177	−0.106	−0.310	0.098	0.308	176	−0.103	−0.311	0.106	0.335
HES1 CpG6	198	−0.098	−0.294	0.097	0.325	197	−0.098	−0.296	0.099	0.330
HES1 CpG7	197	−0.050	−0.244	0.143	0.611	196	−0.040	−0.237	0.156	0.687
HES1 CpG8	195	−0.050	−0.244	0.144	0.615	194	−0.045	−0.242	0.153	0.659
HES1 CpG9	195	−0.089	−0.284	0.105	0.370	194	−0.088	−0.284	0.109	0.382
IED pre-EDS errors										
HES1 CpG1	185	−0.031	−0.100	0.037	0.372	184	−0.025	−0.095	0.045	0.477
HES1 CpG2	178	0.001	−0.070	0.071	0.980	177	0.004	−0.067	0.075	0.912
HES1 CpG3	178	0.014	−0.057	0.086	0.691	177	0.013	−0.058	0.084	0.720
HES1 CpG4	177	−0.028	−0.099	0.043	0.439	176	−0.020	−0.096	0.052	0.581
HES1 CpG5	177	−0.059	−0.127	0.010	0.094*	176	−0.060	−0.129	0.010	0.095*
HES1 CpG6	198	−0.019	−0.085	0.048	0.583	197	−0.016	−0.084	0.051	0.636
HES1 CpG7	197	−0.014	−0.080	0.052	0.678	196	−0.014	−0.081	0.053	0.685
HES1 CpG8	195	−0.047	−0.113	0.019	0.166	194	−0.046	−0.114	0.021	0.182
HES1 CpG9	195	−0.030	−0.097	0.037	0.381	194	−0.028	−0.095	0.040	0.420
SSP-span length										
HES1 CpG1	179	0.086	−0.039	0.211	0.180	178	0.080	−0.047	0.206	0.218
HES1 CpG2	172	0.078	−0.046	0.202	0.221	171	0.066	−0.059	0.191	0.303
HES1 CpG3	172	0.043	−0.082	0.169	0.497	171	0.035	−0.090	0.159	0.584
HES1 CpG4	171	0.083	−0.041	0.207	0.190	170	0.079	−0.046	0.204	0.218
HES1 CpG5	171	0.168	0.048	0.287	0.007**	170	0.149	0.028	0.270	0.017**
HES1 CpG6	192	0.135	0.017	0.253	0.026**	191	0.124	0.005	0.242	0.042**
HES1 CpG7	191	0.144	0.027	0.262	0.017**	190	0.130	0.012	0.249	0.033**
HES1 CpG8	189	0.111	−0.008	0.230	0.069*	188	0.094	−0.027	0.215	0.128
HES1 CpG9	189	0.103	−0.017	0.222	0.095*	188	0.092	−0.029	0.213	0.137

*P-value < 0.1; **P-value ≤ 0.05. CANTAB, Cambridge Neuropsychological Test Automated Battery; LC, lower 95% confidence limit; UCL, upper 95% confidence limit

### Association of cord *HES1* methylation and infant externalising behaviour in the GUSTO cohort

Because of the strong associations found between methylation of specific CpGs within the promoter region of *HES1* and later cognitive function*,* we examined a mediating pathway for this relationship within the Singaporean GUSTO cohort and investigated whether methylation was related to socio-emotional difficulties at an earlier developmental stage, as socially disruptive behaviours have been linked with inattention and a reduced ability to learn. We specifically wanted to test the a priori hypothesis that the DMROI CpGs significantly associated with measures of cognition in the SWS cohort were also associated with measures of emotional regulation. We therefore examined the methylation status of HES1 CpGs 2, 5 and 7 in relation to externalising behaviour in the GUSTO cohort. Adjusting for the child’s sex and maternal educational attainment, higher cord DNA *HES1* CpG7 methylation was associated with a lower infant externalising score at age 1 year (β = −0.068, *P* = 0.02; Table 3, Figure 3d); CpG5 methylation had no association with externalising score, whereas higher CpG2 methylation had a borderline association with higher externalising score (β = 0.053, *P* = 0.05; Table 3).

**Table 3.** Association of umbilical cord *HES1* CpG methylation within the identified DMROI with 1-year externalising in 108 children

	1 year externalising,adjusted for child’s sex					1 year externalising, adjusted for child’s sex and mother’s educational attainment				
	*n*	β	LCL	UCL	*P*-value	*n*	β	LCL	UCL	*P*-value
*HES1*
CpG2	108	0.064	0.011	0.118	0.019*	95	0.053	0.000	0.110	0.050*
CpG5	107	0.025	−0.030	0.079	0.375	94	0.025	−0.031	0.081	0.381
CpG7	108	−0.063	−0.119	−0.006	0.031*	96	−0.068	−0.124	−0.007	0.020*

LCL, lower 95% confidence limit; UC, upper 95% confidence limit.

*P-value ≤ 0.05.

### Functional significance of altered CpG methylation

**Figure 4.**
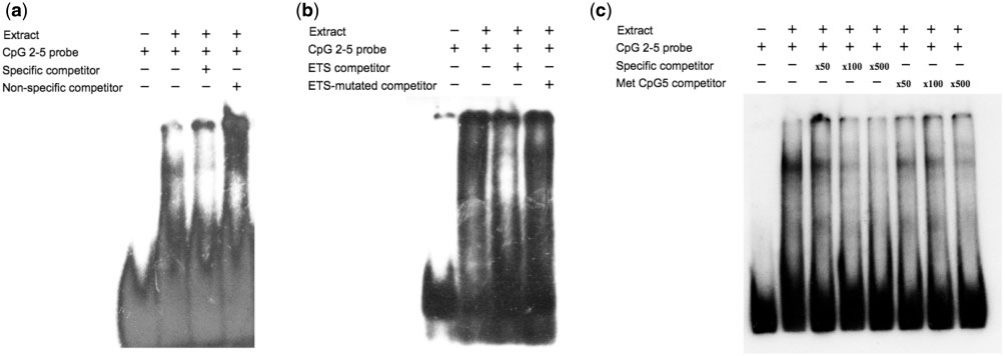
Methylation of CpG5 blocks ETS transcription factor binding to the *HES1* promoter sequence. Results are typical of three analyses. (a) The unmethylated biotin-labelled probe showed a strong shift upon incubation with nuclear extract from the human neuroblastoma cell line IMR32; this shift was markedly reduced by co-incubating with 500-fold excess of the unlabelled specific competitor, but not with 500-fold excess of an unlabelled non-specific competitor. (b) Binding to the probe was markedly diminished by co-incubation with 100-fold excess of an unlabelled oligonucleotide containing the core consensus sequence for ETS (GGAA) but not with 100-fold excess of a mutated ETS core competitor (c) The unmethylated probe was incubated with 50-, 100- and 500-fold excesses of the unmethylated or methylated competitor; binding to the unmethylated probe was competed out with a 100-fold excess of the methylated competitor compared with a 500-fold excess of the unmethylated competitor.

To determine whether methylation of these CpG loci had functional consequences by influencing transcription factor binding to the *HES1* promoter, electrophoretic mobility shift assays (EMSAs) were used. In the human neuroblastoma cell line IMR32, one specific protein complex bound to the *HES1* promoter −4706 to −4740 region, containing CpG sites 2–5 (Figure 4a). *In silico* analysis of this region of the *HES1* promoter using the *Predict transcription factor binding sites* (PROMO) software^31^ predicted that CpG5 was located within an ELK1 (part of the ETS family) binding site. Multiplexed consensus competitor EMSAs^32^ identified the transcription factor bound at this site as part of the ETS transcription factor family. This was confirmed by specific competitive binding with an ETS consensus sequence but not an oligonucleotide containing a mutated core ‘GGAA’ ETS binding sequence (Figure 4b). Moreover, wheras binding was substantially reduced in the presence of 100-fold excess of unmethylated specific competitor, it was unaffected in the presence of 100-fold excess of a specific competitor sequence containing methylated CpG5 (Figure 4c). This suggests that ETS binds preferentially to the unmethylated sequence upstream of *HES1* and methylation of CpG5 inhibits ETS binding to this locus.

## Discussion

There has been much debate regarding the contribution of fixed genetic sequences to variation in IQ in the population, and genome-wide association studies have consistently failed to detect specific SNPs which are associated with a substantial effect.^33^ Here we examined whether perinatal epigenetic processes contribute to cognitive development and function. We show for the first time that methylation of CpG loci in umbilical cord DNA at birth is associated with later neuropsychological outcomes. This provides novel evidence for the importance of developmental epigenetic processes in influencing later cognitive function. Using an MBD array, we identified 41 DMROIs at birth associated with WPPSI IQ at age 4 years. These DMROIs were associated with genes which have been previously linked to cognitive development or function such as *TCF4* (transcription factor 4), a bHLH transcription factor deleted in Pitt-Hopkins syndrome^34^ where individuals exhibit severe motor dysfunction and mental retardation; *IL1RN* (interleukin 1 receptor antagonist) and *MMP3* (matrix metallopeptidase 3), where SNPs associated with these genes have been linked to cognitive decline;^35^^,^^36^ and *NFE2L2* (nuclear factor erythroid 2-like 2) which is known to be decreased in the brain during oxidative stress.^37^ Other genes containing DMROIs such as *FANK1* (fibronectin type III and ankyrin repeat doman 1), *FAM83F* (family with sequence similarity 83 member F) and *SERPINH1* (serpin peptidase inhibitor clade H) have not previously been linked to cognitive function.

Gene ontology analysis of the DMROIs revealed that the top GO process enriched among the DMROIs was diencephalon development. Four genes (*ETS1*, *HES1*, *TCF4* and *NR4A2*) in the diencephalon network contained DMROIs and were included in a direct interactors sub-network. The diencephalon is a region of the brain that functions as a crucial relay and integration centre and modulates sensory, motor and cognitive functions.

Consistent with the findings from the MBD array, sodium bisulphite pyrosequencing in a larger number of SWS subjects confirmed that higher perinatal methylation of CpGs within the *HES1* DMROI correlated with higher IQ at 4 years; adjusting for maternal IQ strengthened the associations between *HES1* methylation and child’s IQ. Higher methylation of *HES1* CpGs 1, 5, 6 and 7 was also associated with higher executive function in an independent group of SWS children at 7 years, including measures of a better visual working memory, greater working memory capacity and increased proficiency in retaining selective attention (assessed by CANTAB DMS and SSP); CANTAB IED outcomes, assessing the ability to engage in deliberate, goal-directed thought and action, were associated with *HES1* CpG8 methylation and there were non-significant trends observed between IED outcomes and methylation of *HES1* CpGs 4, 5, 6 and 9. However, there were differences in the associations found within the WPPSI and CANTAB measurements, for example the methylation of CpGs 5 and 7 was associated with both child’s WPPSI IQ at 4 years and executive function at 7 years, whereas the methylation of CpG2 was only associated with child’s IQ at 4 years and not replicated in relation to executive function at 7 years of age. These differences may reflect different CpG loci within the DMORI having particular effects at different times during development (the cognitive function tests were carried out at different ages), or that the WPPSI and CANTAB tests measure related but different aspects of cognitive function. The DMROI of *HES1* does span a region of over 200 bp and it will be interesting to determine the precise role that these different CpG sites play in the temporal and spatial regulation of *HES1* expression.

To explore the pathway linking *HES1* methylation to later cognitive function, we also examined whether methylation of *HES1* was related to socio-emotional difficulties at an earlier developmental stage, as socially disruptive behaviours have been linked with inattention and a reduced ability to learn.^38^ Interestingly, higher *HES1* CpG7 methylation was also associated with lower externalising scores at age 1 year in the independent GUSTO cohort, suggesting a possible mediating pathway between *HES1* methylation, emotional regulation and eventual cognitive ability, with the lower externalising scores reflecting a decrease in socially disruptive behaviours, consistent with the associations seen between higher *HES1* methylation and increased cognitive function at later ages. Alternatively, *HES1* methylation may impact on neural function to result in both poor emotion regulation and accompanying externalising behaviour as well as cognitive difficulties. In contrast, we observed a borderline association between higher CpG2 methylation and greater externalising. Further replication of the association between *HES1* methylation and socio-emotional behaviour will be required to confirm the direction of the association and whether there is a differential effect of methylation at the different CpG loci within this region on behaviour. It would also be beneficial to examine both externalising and cognitive function outcomes in the same population of children, which may be possible in the GUSTO cohort as the children get older, in order to clarify the potential mediation by emotional regulation on *HES1* methylation and cognitive function.

*HES1* is an effector of the NOTCH signalling pathway that is essential for neural development and function.^39^ Disruption of *Notch1* signalling in *Drosophila* blocks memory consolidation.^40^^,^^41^ Moreover, mice with antisense-reduced hippocampal *Notch1* mRNA and protein levels fail to sustain long-term neural potentiation.^42^ *HES1*, which was originally isolated as a mammalian homologue of *hairy* and *Enhancer of Split*, is an essential mediator of Notch function.^43–45^ Loss and gain of function studies in mice show that *Hes1* is crucial for generating the correct numbers and full diversity of neurons and glial cells by maintaining neural stem cells until later stages through repression of proneural bHLH differentiation factors such as *Mash1* and *Ngn2.*^46^^,^^47^

The DMROI region in *HES1* associated with later cognitive function lies 4.8 kb upstream of the TSS in the *HES1* gene; this is a region highly conserved between species.^48^ DNaseI hypersensitive sites and H3K27 acetylation have also been localised to this region in both embryonic stem cells and neuronal cell lines [http://www.genome.ucsc.edu/ENCODE/], marks associated with active enhancer elements.^49^ Methylation of CpGs within the promoter or regulatory regions of genes is generally thought to block transcription factor binding and/or lead to the recruitment of methyl-binding proteins that in turn recruit histone deacetylases to the DNA, silencing gene expression.^50^^,^^51^ We found that methylation of CpG5, one of the CpGs most strongly associated with later neuropsychological function, blocked binding of an ETS transcription factor to this region. The ETS family of transcription factors comprises 30 different members, which play key roles in ontogenic processes^52^ including the development of the diencephalon, as does *HES1*. Interestingly, the MBD array also identified a DMROI within the ETS1 promoter that was associated with later cognitive function, suggesting that the interplay between these two factors may be important for cognitive development. The reciprocal relationship between *HES1*/*ETS* methylation is consistent with the results of the molecular studies showing that *HES1* methylation blocks ETS binding at the *HES1* promoter. ETS proteins initially contact DNA as a monomeric factor, but they can also form homo- or hetero-dimers with other ETS proteins and/or interact with accessory proteins; dependent upon these interactions they can act as activators or repressors of gene expression.^52^ Thus the effect of inhibiting ETS binding by methylation of specific CpGs within the promoter of *HES1* is likely to be both cell type- and developmental stage-specific. This demonstration that altered methylation can affect transcription factor binding *in vitro* does suggest that methylation at these CpG loci may have functional consequences and potential implications for neuronal development and function. However, a prenatal exposure may affect both *HES1* methylation and neurocognitive outcomes through independent pathways, and the methylation change observed may not directly lead to altered neurocognitive function. Further work is required to establish whether altered methylation of this region of *HES1* is causally involved in neurocognitive development.

To date, genome-wide association studies have identified mutations in *HMGA2* as having the largest impact on IQ; sequence variation within *HMGA2*, however, only alters IQ by 1.29 points.^53^ Here we find that a one SD change in *HES1* methylation is associated with a difference in IQ score of 3.2 points at age 4 years, after controlling for the influences of gender and maternal IQ. These findings suggest that the early life environment operating through epigenetic mechanisms also makes an important contribution to subsequent variation in IQ. It has been shown that the peak enrichment for the distance between CpG and SNPs that are part of *cis*-acting methylation quantitative trait loci (mQTLs) is 45 bp from the CpG site in question.^54^ In our subjects, the presence of SNPs at the identified CpG sites in *HES1* was excluded by direct sequencing of this region, but without genome-wide analysis it is not possible to exclude a genetic effect of distant SNPs which could influence the DNA methylation of a particular sequence.

There are a number of limitations to this study: first, in terms of the methylome approach we used. We measured methylation differences at birth using an MBD array. This has some advantages over the Illumina HumanMethylation450K BeadChip array in terms of greater coverage of the CpG sites within the genome, but MBD capture is biased towards heavily methylated CpG-rich regions. Moreover as the methylated DNA was hybridised to a Human Agilent promoter array, this limits the analysis to CpG sites located within regions relatively close to the TSS of a gene. Thus changes in DNA methylation outside this region will be missed. However, studies from both animal and humans have shown that many environmentally modifiable CpGs sites are located within the promoter regions of genes.^55–58^ We also used a region-centric approach to identify DMROIs; this increases the likelihood of functionally relevant findings, but it comes at the expense of minimising information from the smaller regions of differential methylation. Stringent cut-offs were used to select DMROIs in order to prioritise genes for the pathway analysis, but nonetheless it is likely that the list will include false-positives. The pathway analysis returned a network at a significance level which survived correction for multiple testing, suggesting it includes true-positives; the functionally linked candidate *HES1* picked from this pathway went on to independently replicate. We also measured methylation in cord tissue at birth and it is possible that differences in *HES1* methylation may reflect differences in cellular heterogeneity within the cord tissue but, even if this were the case, these studies still show that altered methylation of cord *HES1* at birth is an effective marker of later neurocognitive function. Recent data have shown that for some genomic regions methylation appears largely independent of tissue of origin, whereas for others there is a clear tissue-specific dependence.^59^ For instance, differential GR methylation in relation to childhood adversity was observed both in peripheral blood and the hippocampus.^11^ It would be interesting to determine whether *HES1* methylation is associated with neurocognitive function in other perinatal tissues or in peripheral blood at later ages, and whether the same assocation between *HES1* methylation and cognitive function is observed also in brain tissue. A further limitation arises from the challenges in assessing neuropsychological function at different ages. IQ cannot be measured in infants, and executive function is widely recognised to be the most important measure of neuropsychological function but cannot easily be assessed in infants and very young children. As a consequence we used different tests at different ages.

## Conclusions

The associations between CpG methylation and neuropsychological function were found in children whose birthweight lay within the normal range and in two culturally diverse populations. The finding of a consistent association between *HES1* methylation at birth and later measures of neuropsychological function suggest that epigenetic processes are important in the regulation of genes and pathways involved in neuropsychological development. However, our data are only correlative and can only imply an association between *HES1* DMROI methylation at birth and later cognitive function. Nevertheless, even if it is a non-causal association, the differential methylation of *HES1* provides an objective marker of an altered developmental trajectory at birth. This has important implications for policy makers and health professionals and strongly supports the growing emphasis on the quality of early life environment not only for optimal short-term health outcomes but also for longer health and well-being.

## Supplementary Data

Supplementary data are available at *IJE* online.

## Funding

This work was supported by grants from the UK Medical Research Council (MC_U147585827, MC_ST_U12055), British Heart Foundation (RG/07/009), Arthritis Research UK, National Osteoporosis Society, International Osteoporosis Foundation, Cohen Trust, Gravida-National Research Centre for Growth and Development, Abbott Nutrition, National Institute for Health Research Musculoskeletal Biomedical Research Unit, University of Oxford and National Institute for Health Research Southampton Biomedical Research Centre, University of Southampton and University Hospital Southampton NHS Foundation Trust, Singapore National Research Foundation and Agency for Science Technology and Research (NMRC/TCR/004-NUS/2008). The funders had no role in study design, data collection and analysis, decision to publish or preparation of manuscript.

**Conflict of interest:** K.G., P.G., C.C. and Y.S.C have received travel reimbursement for speaking at conferences sponsored by companies selling nutritional and pharmaceutical products. The research groups involved in this work are part of an academic consortium that has received funding from Abbott Nutrition, Nestec and Danone.

## Supplementary Material

Supplementary Data
