# Supplementary material for: Association between perinatal methylation of the neuronal differentiation regulator HES1 and later childhood neurocognitive function and behaviour
Source: Int J Epidemiol. 2015 Apr 22;44(4):1263–76. doi: 10.1093/ije/dyv052 (PMC4588869; doi:10.1093/ije/dyv052)
Supplement: Supplementary Data [file supp_44_4_1263__index.html]

Association between perinatal methylation of the neuronal differentiation regulator HES1 and later childhood neurocognitive function and behaviour — Association between perinatal methylation of the neuronal differentiation regulator HES1 and later childhood neurocognitive function and behaviour — Supplementary Data 

# Association between perinatal methylation of the neuronal differentiation regulator *HES1* and later childhood neurocognitive function and behaviour

## Supplementary Data

files

**Files in this Data Supplement:**

- Supplementary Data - docx file
