# Supplementary material for: Association between perinatal methylation of the neuronal differentiation regulator HES1 and later childhood neurocognitive function and behaviour
Source: Int J Epidemiol. 2015 Apr 22;44(4):1263–76. doi: 10.1093/ije/dyv052 (PMC4588869; doi:10.1093/ije/dyv052)
Supplement: Supplementary Data [file supp_dyv052_dyv052Supplementary_Information.docx]

| **Characteristic** | **% or Median (5^th^,95^th^ percentile) for four-year SWS cohort** | **% or Median (5^th^, 95^th^ percentile) for seven-year SWS cohort** |
| --- | --- | --- |
|  |  |  |
| **Mother** |  |  |
| Full Scale IQ (WASI) | 108 (90 to 126) |  |
| Educational qualifications (%) |  |  |
| None | 1.1% | 1.0% |
| CSE | 10.8% | 10.8% |
| O levels | 24.4% | 27.6% |
| A levels | 30.8% | 35.0% |
| HND | 8.5% | 5.4% |
| Degree | 24.4% | 20.2% |
| Social class (%) |  |  |
| Professional | 5.1% | 5.5% |
| Management and technical | 41.8% | 37.2% |
| Skilled non-manual | 34.9% | 34.7% |
| Skilled manual | 8.0% | 6.0% |
| Partly skilled | 9.1% | 14.6% |
| Unskilled | 1.1% | 2.0% |
| Primiparous | 49.4% | 53.4% |
| Age at birth, years | 30.4 (24.5 to 35.6) | 32.2 (25.5 to 36.9) |
| Smoker | 25.0% | 34.4% |
| BMI | 24.5 (20.6 to 33.8) | 24.3 (19.2 to 33.4 ) |
|  |  |  |
| **Child** |  |  |
| Age at follow-up, years | 4.4 (4.2 to 4.6) | 7.0 (6.8 to 7.2) |
| Female (%) | 44.3% | 51.5% |
| Birth order (%) |  |  |
| 1^st^ | 49.4% | 53.4% |
| 2^nd^ | 36.9% | 33.8% |
| 3^rd^ or higher | 13.7% | 12.8% |
| Birth weight, kg | 3.5 (2.7 to 4.4) | 3.4 (2.6 to 4.3) |
| Gestational age, weeks | 40.0 (36.7 to 41.9) | 40.1 (36.9 to 42) |
| Full Scale IQ (WPPSI) | 110 (85 to 127) |  |
| CANTAB assessments |  |  |
| DMS total correct (12s) |  | 3 (1 to 5) |
| IED total errors (stage 1) |  | 2 (0 to 4) |
| IED total errors (stage 8)  IED pre-EDS errors  SSP span length |  | 23 (2 to 31)  8.5 (5 to 23)  4 (2 to 5) |

**Supplementary Table 1: Characteristics of the SWS study participants with four-year (n=175) and seven-year (n=200) cognitive and neuropsychological assessment.** Median birth weight (3.4 kg), maternal age (30.8 years) and pre-pregnancy body mass index (24.1 kg/m^2^) were similar in the whole SWS cohort (n=3,159). WPPSI = Wechsler Pre-School and Primary Scale of Intelligence (full-scale IQ), WASI = Wechsler Abbreviated Scale of Intelligence (full-scale IQ), CANTAB = Cambridge Neuropsychological Test Automated Battery.

| **Characteristic** | **% or Median (5^th^, 95^th^ percentile) for one-year GUSTO cohort** |
| --- | --- |
|  |  |
| **Mother** |  |
| Maternal Highest Education % |  |
| None/Primary | 4.6% |
| Secondary/Technical Education | 22.7% |
| GCE A level/Polytechnic  University/Others | 41.8%  30.9% |
| Ethnicity % |  |
| Chinese | 58.9% |
| Malays | 25.0% |
| Indians | 16.1% |
| Household Monthly Income (SGD) % |  |
| 0-1999 | 15.4% |
| 2000-5999 | 59.1% |
| >6000 | 25.5% |
| Primiparous | 51.6% |
| Age at birth, years | 31.7 (22.7 to 39.4) |
| Smoker | 13.7% |
| BMI at 26 weeks | 25.1 (20.5 to 33.2) |
|  |  |
| **Child** |  |
| Female % | 50.0% |
| Birth order % |  |
| 1^st^ | 51.6% |
| 2^nd^ | 30.7% |
| 3^rd^ or higher | 17.7% |
| Birth weight, kg | 3.09 (2.40 to 3.85) |
| Gestational age, weeks | 38.7 (37.0 to 40.4) |
| Age, years | 0.99 ( 0.92 to 1.12) |
|  |  |
| ITSEA Externalising score | 0.59 (0.13 to 1.04) |
|  |  |
|  |  |

**Supplementary Table 2:** **Characteristics of the 124 GUSTO study participants with one-year neuropsychological assesment.** Median birth weight (3.08 kg), maternal age (30.6 years) and pre-pregnancy body mass index (26.1 kg/m^2^) were also similar in the whole GUSTO cohort (n=1162). ITSEA = Infant Toddler Socio-Emotional Assessment.

| **Primers for bisulphite pyrosequencing** | | | | | |
| --- | --- | --- | --- | --- | --- |
| **Gene** | **Primer** | **Sequence (5’-3’)** | **Genomic co-ordinates (UCSC, hg19, Feb 2009 assembly)** | **Amplicon length (bp)** | **No. CpGs** |
| *HES1* | Forward  Reverse †  Sequencing 1  Sequencing 2 | AGGGGATAAAGGGGAGTT  TCACTTCTTTAATCCCCCTATAACACCA  GGTTTGAAAGTAAATAGGT  TTGTGGGTGGAGATAA | Chr3:193849141-193849361 + | 221 | 9 |
| **Primers for EMSAs** | | | | | |
|  | **Primer** | **Sequence (5’-3’)** |  | | |
| *HES1* | HES1 CpG2-5 | AGTCGCCCTTCCGGGGCGGGGGTGGGGGGACGCTG |  |  |  |
|  | HES1 CpG2-5 methylated2 | AGT*[5MedC]*GCCCTTCCGGGGCGGGGGTGGGGGGACGCTG |  |  |  |
|  | HES1 CpG2-5 methylated5 | AGTCGCCCTTCCGGGGCGGGGGTGGGGGGA*[5MedC]*GCTG |  |  |  |
| *ETS* | Consensus sequence  Mutated consensus | GGGCTGCTTGAGGAAGTATAAGAAT  GGGCTGCTTGAAAAAGTATAAGAAT |  |  |  |

**Supplementary Table 3:** **Primers for bisulphite pyrosequencing and electrophoretic mobility shift assays**. † denotes biotinylated primer.

**Supplementary Table 4:** **Genes containing differentially methylated regions of interests (DMROIs) identified from the MBD array using Fisher Exact tests, sorted by Fisher Exact test p-value.**

| **Chr** | **Gene symbol** | **Fisher Exact**  **p-value** |
| --- | --- | --- |
| *22* | *FAM83F* | 1.00E-06 |
| *1* | *OR6F1* | 1.00E-06 |
| *11* | *SERPINH1* | 1.00E-06 |
| *12* | *BRI3BP* | 1.00E-06 |
| *10* | *FANK1* | 2.00E-06 |
| *17* | *MPP3* | 6.00E-06 |
| *5* | *CMBL* | 7.00E-06 |
| *21* | *C21orf49* | 7.00E-06 |
| *11* | *C11orf61* | 1.50E-05 |
| *8* | *TMEM65* | 2.20E-05 |
| *2* | *IL1RN* | 3.30E-05 |
| *2* | *SLC35F5* | 3.80E-05 |
| *1* | *OR6N2* | 4.00E-05 |
| *2* | *NFE2L2* | 5.00E-05 |
| *7* | *TTC26* | 5.30E-05 |
| *20* | *EYA2* | 7.40E-05 |
| *18* | *TCF4* | 7.80E-05 |
| *3* | *HES1* | 9.60E-05 |
| *12* | *GAS2L3* | 0.000104 |
| *1* | *ZMPSTE24* | 0.000158 |
| *2* | *CIB4* | 0.000325 |
| *8* | *LONRF1* | 0.000422 |

| **Chr** | **Gene symbol** | **Fisher Exact**  **p-value** |
| --- | --- | --- |
| *12* | *KLRC4* | 0.001545 |
| *11* | *RTN3* | 0.001821 |
| *10* | *FGFBP3* | 0.00213 |
| *2* | *RNF103* | 0.002676 |
| *7* | *INHBA-AS1* | 0.003401 |
| *2* | *CIAO1* | 0.004382 |
| *12* | *CLEC12A* | 0.004732 |
| *21* | *SIM2* | 0.005469 |
| *10* | *ACADSB* | 0.005942 |
| *9* | *C9orf5* | 0.006332 |
| *17* | *ABCA5* | 0.006665 |
| *2* | *FER1L5* | 0.007339 |
| *12* | *DERA* | 0.007395 |
| *17* | *FGF11* | 0.008121 |
| *11* | *ETS1* | 0.008175 |
| *4* | *GAB1* | 0.008434 |
| *19* | *CNN1* | 0.009007 |
| *2* | *NR4A2* | 0.00962 |
| *11* | *OR1S1* | * |
|  |  |  |
|  |  |  |
|  |  |  |

*The *OR1S1* region contained on the array included just one 100nt region so it was not possible to conduct a fisher’s exact test. However, that 1 region passed the criteria for a DMR

**Supplementary Table 5: Gene Ontology Processes enrichment analysis of the DMROIs (ontologies with p<0.0005).**

| **GO Process** | **p value** | **Ratio (pathway genes including a DMROI / all genes in pathway** | **DMROIs in each ontology** |
| --- | --- | --- | --- |
| Diencephalon development | 4.430E-05 | 4/71 | *NR4A2, HES1, TCF4, ETS1* |
| Pituitary gland development | 1.88E-04 | 3/40 | *TCF4, HES1, ETS1* |
| Negative regulation of glial cell proliferation | 2.153E-04 | 2/8 | *HES, RNF10* |

|  | **Beta** | **Std. Err** | **t-value** | **p-value** | **LCL** | **UCL** |
| --- | --- | --- | --- | --- | --- | --- |
| **HES1 CpG2** | 2.954 | 1.048 | 2.82 | 0.005 | 0.883 | 5.026 |
| **Sex** | 4.201 | 2.048 | 2.05 | 0.042 | 0.153 | 8.248 |
| **Mother’s WASI** | 0.241 | 0.090 | 2.68 | 0.008 | 0.063 | 0.419 |
| **Parity** | -4.228 | 1.988 | -2.13 | 0.035 | -8.157 | -0.298 |
| **Current Smoking** | 0.567 | 2.350 | 0.24 | 0.810 | -4.077 | 5.211 |
| **Birthweight** | 0.003 | 0.002 | 1.36 | 0.175 | -0.001 | 0.007 |
| **Age at test** | -3.834 | 7.857 | -0.49 | 0.626 | -19.362 | 11.693 |
| **Mother’s BMI** | -0.189 | 0.240 | -0.79 | 0.432 | -0.663 | 0.285 |

|  | **Beta** | **Std. Err** | **t-value** | **p-value** | **LCL** | **UCL** |
| --- | --- | --- | --- | --- | --- | --- |
| **HES1 CpG5** | 1.766 | 1.108 | 1.59 | 0.113 | -0.426 | 3.957 |
| **Sex** | 5.976 | 2.160 | 2.73 | 0.007 | 1.643 | 10.310 |
| **Mother’s WASI** | 0.251 | 0.100 | 2.51 | 0.013 | 0.053 | 0.448 |
| **Parity** | -3.474 | 2.161 | -1.59 | 0.115 | -7.809 | 0.861 |
| **Current Smoking** | 1.411 | 2.523 | 0.56 | 0.577 | -3.580 | 6.402 |
| **Birthweight** | 0.002 | 0.002 | 0.92 | 0.360 | -0.003 | 0.007 |
| **Age at test** | -3.256 | 8.524 | -0.38 | 0.703 | -20.123 | 13.611 |
| **Mother’s BMI** | -0.141 | 0.257 | -0.55 | 0.586 | -0.650 | 0.369 |

**Supplementary Table 6: Adjustment covariates for HES1 CpG2**

|  | | **SWS subjects at four- years** | **SWS subjects at**  **seven- years** | **GUSTO subjects** |
| --- | --- | --- | --- | --- |
|  | **Genomic co-ordinates (hg19)**  **and distance from TSS** | Methylation %, median  (5th, 95^th^ percentile) | Methylation %, median  (5th, 95^th^ percentile) | Methylation %, median  (5th, 95^th^ percentile) |
| ***HES1***  **CpG1** | chr3:193849210+ (-4721) | 47.55 (41.4, 53.76) | 45.99 (40.61,52.66) | 47.33 (42.42, 52.04) |
| **CpG2** | chr3:193849227+ (-4704) | 45.16 (39.76, 50.75) | 43.04 (35.76,48.41) | 44.66 (39.60, 48.99) |
| **CpG3** | chr3:193849235+ (-4696) | 35.43 (30.74, 43.23) | 34.29 (29.37,41.57) | 33.70 (29.03, 37.61) |
| **CpG4** | chr3:193849240+ (-4691) | 42.97 (37.64, 48.33) | 42.16 (35.85,50.13) | 42.39 (37.03, 46.98) |
| **CpG5** | chr3:193849254+ (-4679) | 27.64 (21.93, 35.77) | 24.16 (19.05,34.77) | 23.35 (19.34, 28.11) |
| **CpG6** | chr3:193849275+ (-4656) | 32.18 (26.21, 42.12) | 31.20 (26.05,42.16) | 29.04 (24.96, 39.10) |
| **CpG7** | chr3:193849309+ (-4622) | 24.51 (19.13, 31.83) | 20.77 (16.15,28.30) | 21.89 (18.20, 30.24) |
| **CpG8** | chr3:193849318+ (-4613) | 23.02 (17.54, 32.12) | 19.42 (14.71,30.57) | 19.60 (15.62, 29.52) |
| **CpG9** | chr3:193849328+ (-4603) | 32.60 (27.08, 41.59) | 29.12 (23.23,38.74) | 30.69 (26.27, 40.07) |
|  |  |  |  |  |

**Supplementary Table 7**: **Umbilical cord methylation range within the DMROI of *HES1* as measured by bisulphite pyrosequencing in the SWS and GUSTO cohort.**

**Supplementary Table 8**: **Correlation of the differentially methylated HES1 CpGs at age 4- and 7-years in the SWS cohort**

| **Four-year cohort** | | | | | | | | | | |
| --- | --- | --- | --- | --- | --- | --- | --- | --- | --- | --- |
|  |  | **CpG1** | **CpG2** | **CpG3** | **CpG4** | **CpG5** | **CpG6** | **CpG7** | **CpG8** | **CpG9** |
| **CpG1** | **Correlation Coefficient** | 1.000 | .634^**^ | .538^**^ | .510^**^ | .412^**^ | .446^**^ | .666^**^ | .568^**^ | .637^**^ |
|  | **Sig (2-tailed)** |  | .000 | .000 | .000 | .000 | .000 | .000 | .000 | .000 |
|  | **n** | 168 | 157 | 154 | 146 | 139 | 163 | 148 | 140 | 131 |
| **CpG2** | **Correlation Coefficient** | .634^**^ | 1.000 | .611^**^ | .573^**^ | .578^**^ | .589^**^ | .659^**^ | .605^**^ | .669^**^ |
|  | **Sig (2-tailed)** | .000 |  | .000 | .000 | .000 | .000 | .000 | .000 | .000 |
|  | **n** | 157 | 157 | 154 | 146 | 139 | 152 | 137 | 129 | 122 |
| **CpG3** | **Correlation Coefficient** | .538^**^ | .611^**^ | 1.000 | .467^**^ | .571^**^ | .457^**^ | .498^**^ | .503^**^ | .623^**^ |
|  | **Sig (2-tailed)** | .000 | .000 |  | .000 | .000 | .000 | .000 | .000 | .000 |
|  | **n** | 154 | 154 | 154 | 146 | 139 | 149 | 134 | 126 | 120 |
| **CpG4** | **Correlation Coefficient** | .510^**^ | .573^**^ | .467^**^ | 1.000 | .636^**^ | .518^**^ | .644^**^ | .578^**^ | .595^**^ |
|  | **Sig (2-tailed)** | .000 | .000 | .000 |  | .000 | .000 | .000 | .000 | .000 |
|  | **n** | 146 | 146 | 146 | 146 | 137 | 141 | 127 | 120 | 115 |
| **CpG5** | **Correlation Coefficient** | .412^**^ | .578^**^ | .571^**^ | .636^**^ | 1.000 | .691^**^ | .554^**^ | .619^**^ | .647^**^ |
|  | **Sig (2-tailed)** | .000 | .000 | .000 | .000 |  | .000 | .000 | .000 | .000 |
|  | **n** | 139 | 139 | 139 | 137 | 139 | 135 | 121 | 113 | 109 |
| **CpG6** | **Correlation Coefficient** | .446^**^ | .589^**^ | .457^**^ | .518^**^ | .691^**^ | 1.000 | .546^**^ | .560^**^ | .582^**^ |
|  | **Sig (2-tailed)** | .000 | .000 | .000 | .000 | .000 |  | .000 | .000 | .000 |
|  | **n** | 163 | 152 | 149 | 141 | 135 | 170 | 155 | 146 | 138 |
| **CpG7** | **Correlation Coefficient** | .666^**^ | .659^**^ | .498^**^ | .644^**^ | .554^**^ | .546^**^ | 1.000 | .850^**^ | .798^**^ |
|  | **Sig (2-tailed)** | .000 | .000 | .000 | .000 | .000 | .000 |  | .000 | .000 |
|  | **n** | 148 | 137 | 134 | 127 | 121 | 155 | 155 | 146 | 138 |
| **CpG8** | **Correlation Coefficient** | .568^**^ | .605^**^ | .503^**^ | .578^**^ | .619^**^ | .560^**^ | .850^**^ | 1.000 | .808^**^ |
|  | **Sig (2-tailed)** | .000 | .000 | .000 | .000 | .000 | .000 | .000 |  | .000 |
|  | **n** | 140 | 129 | 126 | 120 | 113 | 146 | 146 | 146 | 136 |
| **CPG9** | **Correlation Coefficient** | .637^**^ | .669^**^ | .623^**^ | .595^**^ | .647^**^ | .582^**^ | .798^**^ | .808^**^ | 1.000 |
|  | **Sig (2-tailed)** | .000 | .000 | .000 | .000 | .000 | .000 | .000 | .000 |  |
|  | **n** | 131 | 122 | 120 | 115 | 109 | 138 | 138 | 136 | 138 |
| **Seven-year cohort** | | | | | | | | | | |
|  |  | **CpG1** | **CpG2** | **CpG3** | **CpG4** | **CpG5** | **CpG6** | **CpG7** | **CpG8** | **CpG9** |
| **CpG1** | **Correlation Coefficient** | 1.000 | .636^**^ | .592^**^ | .510^**^ | .490^**^ | .538^**^ | .498^**^ | .420^**^ | .512^**^ |
|  | **Sig (2-tailed)** |  | .000 | .000 | .000 | .000 | .000 | .000 | .000 | .000 |
|  | **n** | 218 | 210 | 210 | 209 | 209 | 218 | 216 | 215 | 215 |
| **CpG2** | **Correlation Coefficient** | .636^**^ | 1.000 | .540^**^ | .488^**^ | .479^**^ | .487^**^ | .455^**^ | .410^**^ | .450^**^ |
|  | **Sig (2-tailed)** | .000 |  | .000 | .000 | .000 | .000 | .000 | .000 | .000 |
|  | **n** | 210 | 210 | 210 | 209 | 209 | 210 | 208 | 207 | 207 |
| **CpG3** | **Correlation Coefficient** | .592^**^ | .540^**^ | 1.000 | .307^**^ | .451^**^ | .497^**^ | .471^**^ | .468^**^ | .581^**^ |
|  | **Sig (2-tailed)** | .000 | .000 |  | .000 | .000 | .000 | .000 | .000 | .000 |
|  | **n** | 210 | 210 | 210 | 209 | 209 | 210 | 208 | 207 | 207 |
| **CpG4** | **Correlation Coefficient** | .510^**^ | .488^**^ | .307^**^ | 1.000 | .576^**^ | .510^**^ | .455^**^ | .476^**^ | .437^**^ |
|  | **Sig (2-tailed)** | .000 | .000 | .000 |  | .000 | .000 | .000 | .000 | .000 |
|  | **n** | 209 | 209 | 209 | 209 | 209 | 209 | 207 | 206 | 206 |
| **CpG5** | **Correlation Coefficient** | .490^**^ | .479^**^ | .451^**^ | .576^**^ | 1.000 | .730^**^ | .697^**^ | .697^**^ | .630^**^ |
|  | **Sig (2-tailed)** | .000 | .000 | .000 | .000 |  | .000 | .000 | .000 | .000 |
|  | **n** | 209 | 209 | 209 | 209 | 209 | 209 | 207 | 206 | 206 |
| **CpG6** | **Correlation Coefficient** | .538^**^ | .487^**^ | .497^**^ | .510^**^ | .730^**^ | 1.000 | .735^**^ | .733^**^ | .747^**^ |
|  | **Sig (2-tailed)** | .000 | .000 | .000 | .000 | .000 |  | .000 | .000 | .000 |
|  | **n** | 218 | 210 | 210 | 209 | 209 | 231 | 229 | 227 | 227 |
| **CpG7** | **Correlation Coefficient** | .498^**^ | .455^**^ | .471^**^ | .455^**^ | .697^**^ | .735^**^ | 1.000 | .811^**^ | .828^**^ |
|  | **Sig (2-tailed)** | .000 | .000 | .000 | .000 | .000 | .000 |  | .000 | .000 |
|  | **n** | 216 | 208 | 208 | 207 | 207 | 229 | 229 | 227 | 227 |
| **CpG8** | **Correlation Coefficient** | .420^**^ | .410^**^ | .468^**^ | .476^**^ | .697^**^ | .733^**^ | .811^**^ | 1.000 | .830^**^ |
|  | **Sig (2-tailed)** | .000 | .000 | .000 | .000 | .000 | .000 | .000 |  | .000 |
|  | **n** | 215 | 207 | 207 | 206 | 206 | 227 | 227 | 227 | 227 |
| **CPG9** | **Correlation Coefficient** | .512^**^ | .450^**^ | .581^**^ | .437^**^ | .630^**^ | .747^**^ | .828^**^ | .830^**^ | 1.000 |
|  | **Sig (2-tailed)** | .000 | .000 | .000 | .000 | .000 | .000 | .000 | .000 |  |
|  | **n** | 215 | 207 | 207 | 206 | 206 | 227 | 227 | 227 | 227 |

**. Correlation is significant at the 0.01 level (2-tailed).

**Supplementary Methods 1: The SWS cohort and neurological assesment**

***Southampton Women’s Survey: participants***

The Southampton Women’s Survey (SWS) is a prospective mother offspring cohort study that has assessed the diet, body composition, physical activity and social circumstances of a large group of non-pregnant women aged 20 to 34 years living in the city of Southampton, UK. Women were recruited through General Practices across the city between April 1998 and December 2002. Each woman was invited to take part by letter, followed by a telephone call when an interview date was arranged; 12,583 women agreed to take part, 75% of all women contacted. Trained research nurses visited the women at home and collected information about their health, diet and lifestyles, as well as taking anthropometric measurements. Women who subsequently became pregnant were followed up at 11, 19 and 34 weeks gestation and their offspring were studied in infancy and childhood. Details of mothers’ parity, educational attainment (defined in six groups according to highest academic qualification) and social class were obtained at the pre-pregnancy interview, and height and weight were measured. Amongst women who became pregnant, smoking status in pregnancy was ascertained at the 11 and 34 week interviews. A total of 1981 women became pregnant and delivered a live-born singleton infant before the end of 2003. Six infants died in the neonatal period and two had major congenital growth abnormalities, which left 1973 mother-offspring pairs. Follow-up of the children and sample collection/analysis was carried out under Institutional Review Board approval (Southampton and SW Hampshire Research Ethics Committee) with written informed consent. Investigations were conducted according to the principles expressed in the Declaration of Helsinki.

*SWS cognitive and neuropsychological assessment at seven-years of age*

The Cambridge Neuropsychological Test Automated Battery (CANTAB^®^) is designed to test specific components of executive function. To reduce the likelihood of chance findings we focused on five CANTAB^®^ outcomes with the strongest track record of associations with executive function in the published literature (Delayed Matching to Sample (DMS) 12 s delay total correct, Intra-Extra Dimensional Set Shift (IED) Stage 1 errors, IED Pre- extra-dimensional shift (EDS) errors, IED Stage 8 errors, Spatial Span (SSP) span length). DMS assesses forced choice recognition memory for novel non-verbalisable patterns, and tests both simultaneous and short term visual memory; 12 s delay DMS total correct ranged between 0 and 5, with a median of 3. IED involves a total of nine stages and assesses the ability to engage in deliberate, goal-directed thought and action. The number of errors committed on stage 1 indicates proficiency in detecting and learning the implicit rule of the task based on feedback from the experimenter as to whether the choice was correct. The total number of errors from Stages 1 to 7 is referred to as Pre-EDS errors and indicates proficiency in maintaining selective attention. Successful completion of stages indicates ability to maintain attention and the flexibly to shift in response to the demands of the task. At Stage 8 (the EDS stage) participants must learn to shift attention from the previously correct dimension (the shape of the stimulus) to the newly correct dimension (the line); the number of errors at this stage indicates proficiency in extra-dimensional set-shifting. Two children completed only stage 1 of the task and the data from these participants were removed from the IED analyses. Stage 1 errors ranged from 0 to 23; for statistical analysis the data were analysed in 5 groups. Stage 1-7 errors were positively skewed and were log-transformed for statistical analysis. Stage 8 errors ranged from 0 to 35; for statistical analysis the data was analysed in five groups. SSP assesses the working memory capacity aspect of executive function. White squares are shown, some of which briefly change colour in a variable sequence. The participant must then touch the boxes which changed colour in the same order that they were displayed by the computer (for clinical mode) or in the reverse order (for reverse mode). The number of boxes (and level of difficulty) increases from two at the start of the test to nine at the end, and the sequence and colour are varied through the test. We used the span length (the longest sequence of the pattern the participant is able to follow) as a measure of working memory capacity, which is shorter in children with bipolar disorder. Spatial span length ranged between two and six, with a median of four. Direct assessments of the mother’s cognitive function were not available for analyses relating to this group of seven-year old children; we therefore controlled for the mother’s level of educational attainment as a principal potential confounding factor for child’s cognitive function.

**Supplementary Methods 2: The GUSTO cohort and neurological assesment**

***Growing Up in Singapore Towards Healthy Outcomes (GUSTO): participants***

Mothers were recruited from the KK Women’s and Children’s Hospital and the National University Hospital in Singapore. 3751 families were screened and 2034 met eligibility criteria. Ineligibility was principally accounted for by an intention to deliver outside the 2 study hospitals or not to remain in Singapore for the next 5 years, booking beyond the first trimester, or non-homogenous parental ethnic background. Of the 1247 women (response rate 61.3%) recruited, 1162 conceived naturally, while 85 conceived through in vitro fertilisation (IVF). At baseline, 55.9% were Chinese, 26.1% Malay and 18% Indian. Mean maternal age at recruitment was 30.6 years (range: 18 to 46). Gestational age was defined from a dating ultrasound (10–12 weeks) followed by an additional scan at 18–22 weeks. The average birth weight in the GUSTO cohort was 3081 g, which is comparable to the average across a larger Singaporean sample of 3183 g for a term infant (unpublished data). Written parental consent to participate in the study was given and hard copies are stored by the GUSTO data team. Ethical approval for the study and the consent forms and contents was granted, by the ethics boards of both KKH and NUH, which are centralised Institute Review Board (CIRB) and Domain Specific Review Board (DSRB), respectively.

*GUSTO neuropsychological assessment at one year of age*

The ITSEA was administered to mothers via questionnaire format. The ITSEA detects social-emotional and behaviour problems and delays in the acquisition of competencies in infants and toddlers. It is designed to be applicable to a wide range of parents including those with limited education and from different cultural backgrounds. The Externalising domain considers early manifestations of socially disruptive behaviours such as aggression and defiance. Here, we include ITSEA data from the 124 GUSTO children for whom umbilical cord DNA was also available.

**Supplementary Methods 3. Whole Genome methylation analysis**

After methyl capture, the labelled methylated DNA and input DNA was hybridised to the Agilent Human Promoter Whole-Genome ChIP-on-chip array (G4489A). This contains probes which are split across 2 plates, one accommodating chromosomes 1 to 10 and the other accommodating chromosomes 10/11 to 22 together with X and Y. Microarray hybridisation of the methylated DNA and input DNA (sonicated, total DNA) was carried out by Oxford Gene Technology (OGT, Oxford UK) in accordance with the company’s quality control procedures using standard protocols for labelling, hybridisation and washing. Microarray slides were scanned at 5μM resolution using the extended dynamic range (high 100%, low 10%). The slides were then feature extracted using Agilent Feature Extraction Software (v.9.5.3.1). All arrays were normalised per spot and per chip by an intensity dependent normalisation (Lowess normalisation) using Agilent Genespring Software.

A minority of samples from each plate (most of which were technical replicates), deviated from the expected beta distribution and were discarded. 22 samples for plate one and 21 samples for plate two, remained. All the remaining samples had median absolute deviation (MAD) scores above -5 and did not cluster into strongly defined separate groups in unsupervised hierarchical clustering or principal component analysis.
